# Supplementary material for: Fabry Disease and Central Nervous System Involvement: From Big to Small, from Brain to Synapse
Source: Int J Mol Sci. 2023 Mar 9;24(6):5246. doi: 10.3390/ijms24065246 (PMC10049671; doi:10.3390/ijms24065246)
Supplement: Supplementary file 1 [file ijms-24-05246-s001.zip › ijms-2206806-supplementary.pdf]

SUPPLEMENTARY INFORMATIONS

1.METHODS

Different references encompassing general knowledge on the Fabry pathophysiology, energy metabolism in different neurometabolic disorders or general concepts on lysosomal storage disorders were also revised for the present work. Nevertheless, the specific search strategy for addressing Fabry SNC involvement (other than stroke) has been conducted as specified in the next lines.

1. Search Strategy

Search terms were developed on the basis of Medical Subject Headings (MeSH), and free-text words and abbreviations related to Fabry disease with respect to neuropsychiatric disorders, brain MRI abnormalities, animal and cellular models, neuronal or neurotransmission abnormalities, and Parkinsonism/neurodegeneration. The specific search terms and Boolean operators' strategy were as follows: (addiction OR depression OR neuropsychiatric OR suicide OR cognitive OR "brain MRI" OR "animal model" OR "cellular model" OR Parkinsonism OR neurodegeneration OR neurotransmission OR "neuronal dysfunction) AND Fabry.

The databases searched were the US National Library of Medicine (includes PubMed and MEDLINE), and Embase. The searches were conducted in September 2021, with restrictions in order to include only the more recent references published from 2011 to present. Additional references identified from the reference lists of published articles were identified.

2. Eligibility Criteria

Articles in English, French, German and Spanish, with abstract available, were included. All articles identified during the literature searches were screened to select relevant articles for inclusion. Duplicates were manually excluded. Screening was based on the abstract and title. The decision to be included for revision was made based on the prespecified inclusion/exclusion criteria and on the special topic covered by the article.

Subsequently, a hand search of reference lists was performed to screen for other articles worth to be included. Lastly, full-text articles were assessed and included if the topic of interest was relevant and of interest to be mentioned for the review work.

2. RESULTS

As a summary, the screening process accounted for a total of 666 articles that were identified in the literature searches in Embase and PubMed. Following the removal of duplicate records, and those that did not fulfilled the inclusion criteria, 36 articles were screened for inclusion. Hand search of reference lists meant for another 22 articles. The identification, screening and reasons for exclusion of publications are summarized in Figure 1.

Supplementary Figure S1. Flow diagram of identification, screening and inclusion of articles in the review of the literature.

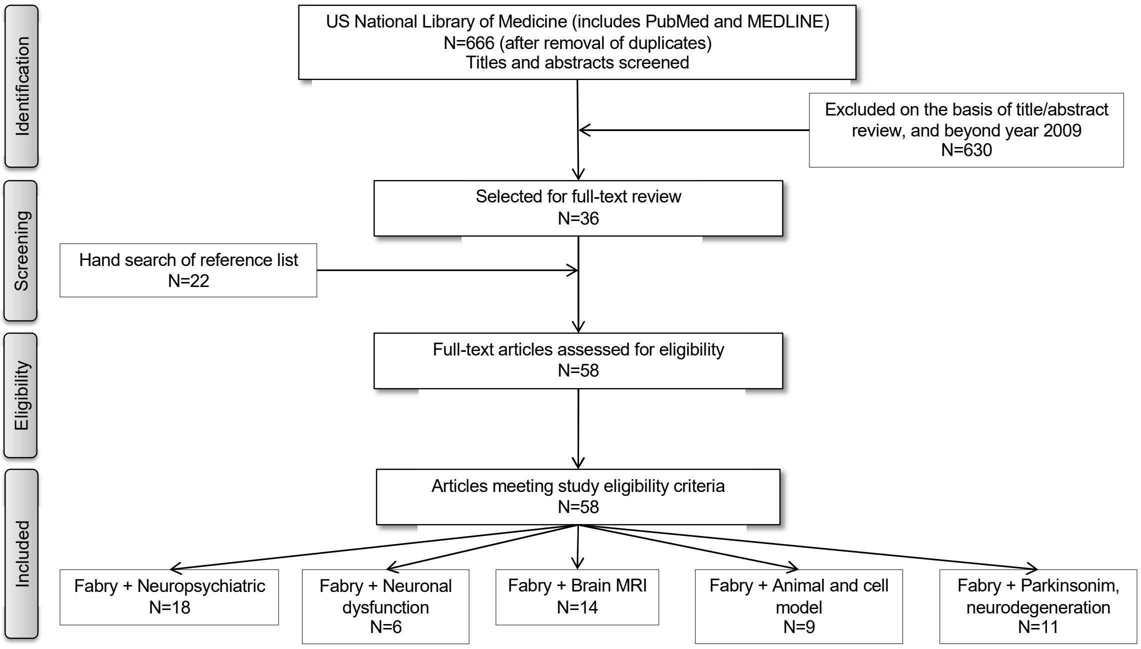

The figure shows the literature identification and study selection process for publications reporting on Fabry disease and its involvement in central nervous system, beyond cerebrovascular disease.

The number of articles found covering each topic, and the ones that were finally accepted for inclusion are summarized in Table S1.

Supplementary Table S1. Summary of the number of articles covering each topic on Fabry disease and every specific central nervous system involvement.

Search term	Number of papers by search	Number of papers selected*	Number of papers added**
Fabry + addiction	4	0	0
Fabry + depression	70	2	1
Fabry + neuropsychiatric	9	2	3
Fabry + suicide	3	1	0
Fabry + cognitive	66	7	2
Fabry + (brain MRI)	181	10	4
Fabry + (cell + animal model)	227	7	2
Fabry + Parkinson + neurodegeneration	36	6	5
Fabry + neurotransmission	3	0	1
Fabry + (neuronal dysfunction)	67	1	4

This table summarizes the number of articles covering each topic on Fabry disease and every specific central nervous system involvement (beyond stroke and neuropatic pain).
*The numbers on the second column indicate the total number of papers included for review after filtering based on abstract content and excluding duplicates.
**Numbers on the last column indicate the number of articles that were considered of interest, and were added after revision of the reference list from other articles.
